# Bisphosphonate Use for Glucocorticoid-Induced Osteoporosis in Elderly Patients with Immune Thrombocytopenia Receiving Prolonged Steroid Therapy: A Single Institute Retrospective Study

**DOI:** 10.3390/hematolrep14030039

**Published:** 2022-09-19

**Authors:** Satoshi Yamasaki, Kenjiro Kamezaki, Yoshikiyo Ito, Takahiko Horiuchi

**Affiliations:** 1 Department of Internal Medicine, Kyushu University Beppu Hospital, 4546 Tsurumihara, Beppu 874-0838, Japan; 2 Department of Hematology and Clinical Research Institute, National Hospital Organization Kyushu Medical Center, Fukuoka 810-0065, Japan; 3 Department of Hematology, National Hospital Organization, Fukuoka Higashi Medical Center, Fukuoka 811-3195, Japan; 4 Department of Hematology, Imamura General Hospital, Kagoshima 890-0064, Japan

**Keywords:** immune thrombocytopenia, glucocorticoid-induced osteoporosis, elderly patients, FRAX^®^, Garvan tool, bisphosphonate

## Abstract

Prednisolone, used as a standard initial treatment for immune thrombocytopenia (ITP), is an important risk factor for osteoporosis. To investigate the prevention of glucocorticoid-induced osteoporosis (GIO) in elderly ITP patients receiving prolonged steroid therapy, associations between GIO prevention and the real-world data of score changes of a dual-energy X-ray absorptiometry (DXA) scan, FRAX^®^ and the Garvan tool during the initial loading of prednisolone were examined. In our institute, 22 ITP patients aged ≥ 70 years received 0.5–1.0 mg/kg prednisolone for 2–3 weeks as the initial ITP treatment between 2014 and 2021. The femoral neck bone mineral density (BMD) measured by DXA scan was entered into FRAX^®^ to define the risk-adapted approach to bisphosphonate during the initial loading of prednisolone. Bisphosphonate was administered according to <−1.0 femoral neck BMD T-score measured by DXA scan. Worse scores of FRAX^®^ and the Garvan tool were associated with bisphosphonate use for short-term fracture prevention in primary GIO; however, there were no incidents of fracture or significant differences in probabilities determined by FRAX^®^ and the Garvan tool. During the initial loading of prednisolone, prescribing bisphosphonate might prevent the reduction in BMD in elderly patients with ITP receiving prolonged steroid therapy.

## 1. Introduction

Glucocorticoid therapy is the standard initial treatment for immune thrombocytopenia (ITP) and is an important risk factor for osteoporosis [1]. In Japan, *Helicobacter pylori* eradication [2] is considered before initial prednisolone therapy for ITP [3,4]. Alterations in immune homeostasis in the setting of other autoimmune diseases have been postulated to cause ITP [5], and a positive antinuclear antibody test can be considered before ITP treatment, especially splenectomy [6].

Glucocorticoids are associated with a risk of bone loss, which is most pronounced in the first few months of use [7]. Recently, strategies that avoid glucocorticoid side effects have been favored, and a strong emphasis has been placed on shared decision-making, especially regarding second-line therapy, including the early administration of thrombopoietin receptor agonist (TPO-RA) [8]. Glucocorticoids increase the risk of fracture, which occurs in the early course of treatment, during the rapid phase of bone loss, and at higher bone mineral density (BMD) levels than those in postmenopausal osteoporosis [9]. An increased risk of fracture was reported for doses of prednisolone as low as 2.5–7.5 mg daily [10]. The increased risk of fracture in patients taking glucocorticoids declined rapidly over one year of therapy [11]. The assessment of fracture risk using the 2017 American College of Rheumatology guidelines requires the evaluation of clinical risk factors for fracture and BMD [11]. The value of BMD is commonly expressed as a T-score. A T-score ≤ −2.5 is consistent with a diagnosis of osteoporosis, a T-score between −1.0 and −2.5 is classified as low bone mass (osteopenia), and a T-score of −1.0 or higher is normal [12]. For patients aged 40–90 years, clinical risk factors with a BMD measurement can be entered into a validated algorithm (FRAX^®^), available online at www.sheffield.ac.uk/FRAX/tool.jsp (accessed on 7 October 2021), which calculates the 10-year probabilities of major osteoporotic (clinical vertebral, hip, humerus, and wrist) and hip fractures. A significant negative correlation between BMD and total and mean daily steroid dose was reported for glucocorticoid-induced osteoporosis (GIO) in adult patients with ITP scheduled to receive long-term steroid treatment and bisphosphonate was an effective agent for the prevention and treatment of GIO [13]. However, currently there are no data on elderly patients with ITP receiving prolonged steroid therapy.

To fill the evident gap in the management of elderly patients with ITP in real-world practice, we conducted a retrospective investigation at our institution. The aim of this study was to investigate the prevention of GIO for elderly ITP patients.

## 2. Materials and Methods

### 2.1. Patient Selection

We retrospectively reviewed the clinical data of patients aged ≥ 70 years who were newly diagnosed with ITP, assessed with platelet counts <20 × 10^9^/L, and treated with initial prednisolone therapy from October 2014 to July 2021 at Kyushu University Beppu Hospital. We excluded patients with a history of malignancy, previous fragility fractures, or those who received radiotherapy.

The examined variables were as follows: (1) patient-related variables including age at diagnosis, sex, antinuclear antibody, history of autoimmune disease, history of *Helicobacter pylori* eradication, and follow-up time; (2) disease-related variables including baseline platelet level, total prednisolone dose for the initial treatment until <5 mg prednisolone daily administration, duration of ≥5 mg prednisolone daily administration, administration of TPO-RA, and the prescription of bisphosphonate and active vitamin D; and (3) outcome variables, including femoral neck BMD T-score and lumbar BMD T-score using a dual-energy X-ray absorptiometry (DXA) scan, FRAX^®^ 10-year probabilities of major osteoporotic and hip fractures, probabilities defined by the Garvan tool during the initial loading of prednisolone treatment, and the serum 25-hydroxyvitamin D (25(OH)D) level at the tapering phases of the course of prednisolone treatment. The enrolled patients were followed until the end of follow-up, which was defined as the end of the post-hospital follow-up visit. Informed consent to participate in the study was obtained by providing patients and their guardians with information about the opt-out form on our hospital website. The study was performed in accordance with the institutional guidelines and the principles of the Declaration of Helsinki. The protocol was approved by the institutional review board.

### 2.2. Treatment

All patients received 0.5–1.0 mg/kg prednisolone for 2 weeks, to a maximum of 3 weeks as the initial treatment for ITP. Dose modification and the timing of the start were at the physician’s discretion. Prednisolone was tapered, aiming to reduce the dose to <5 mg prednisolone daily. TPO-RA was considered for the treatment of patients who had an insufficient response to prednisolone and in whom it was difficult to start tapering prednisolone.

During the initial loading of prednisolone treatment, all patients were assessed for fracture risk using the scoring method: age (≥50 years, score 2; ≥65 years, score 4), prednisolone dose (≥5 mg/day, score 1; ≥7.5 mg/day, score 4), lumbar BMD (<80% young adult mean (YAM), score 2; <70% YAM, score 4), and prior fragility fractures (yes, score 7) were identified as factors predicting future fracture and the fracture risk for an individual was calculated as the sum of the scores for each risk factor according to the Japanese guidelines [14], which were updated on the basis of a score of 3 as the optimal cut-off score for pharmacological intervention. The medications recommended in the guidelines are limited to those approved for the treatment of osteoporosis in Japan [14]. In our institute, bisphosphonate treatment was performed during the initial loading of prednisolone treatment according to a femoral neck BMD T-score < −1.0, which is classified as low bone mass (osteopenia) [12], and active vitamin D with bisphosphonate was prescribed for women. Serum 25(OH)D levels were measured at the tapering phases of the course of prednisolone treatment to evaluate adequate vitamin D levels.

### 2.3. Response Criteria

Responses were judged according to the criteria of the International Working Group [15]. Treatment response was defined as a complete response (CR) with a sustained platelet count of >100 × 10^9^/L without any bleeding tendency. Platelet counts were confirmed on two separate occasions at least 7 days apart when defining CR.

### 2.4. Prescription for the Prevention of Glucocorticoid-Induced Osteoporosis

During the initial loading of prednisolone treatment, all 22 patients underwent a DXA scan before bisphosphonate treatment, and their femoral neck BMD was entered into FRAX^®^ to define the risk-adapted approach to bisphosphonate treatment (*n* = 15), which included alendronate (*n* = 9) and risedronate (*n* = 6). Seven patients who achieved CR received bisphosphonate treatment and four women received active vitamin D prescription. Eight patients with refractory ITP who received TPO-RA also received bisphosphonate treatment and seven women received active vitamin D prescription.

**Figure 1:**
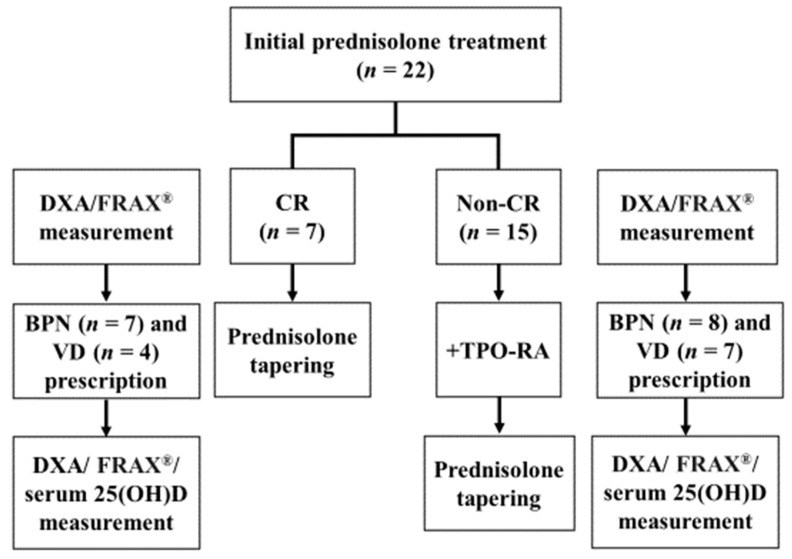
Treatment charts of 22 patients with immune thrombocytopenia. Seven patients achieved a complete response (CR). Fifteen patients had a platelet count <30 × 10^9^/L, all of whom received thrombopoietin receptor agonist treatment. After adding a thrombopoietin receptor agonist, nine patients achieved CR and six patients achieved a response that was less than a CR. The analysis of a dual-energy X-ray absorptiometry scan and measurement of femoral neck bone mineral density were entered into FRAX^®^ to define the risk-adapted approach to bisphosphonate treatment (during the initial loading and tapering phases of prednisolone treatment). Active vitamin D with bisphosphonate was prescribed for women. Serum 25-hydroxyvitamin D levels were measured at the tapering phases over the course of prednisolone treatment to evaluate adequate vitamin D levels. DXA, a dual-energy X-ray absorptiometry; FRAX^®^, a validated algorithm, available online at www.sheffield.ac.uk/FRAX/tool.jsp (accessed on 7 October 2021); BPN, bisphosphonate; VD, active vitamin D; 25(OH)D, 25-hydroxyvitamin D; CR, complete response; TPO-RA, thrombopoietin receptor agonist.

Active vitamin D, including alfacalcidol (*n* = 6) and eldecalcitol (*n* = 5), with bisphosphonate was prescribed for women (*n* = 11). The treatment charts of the 22 patients with ITP are shown in Figure 1.

### 2.5. Statistical Methods

We analyzed the frequencies and descriptive statistics of the patients, disease, and outcome variables in patients with ITP. Continuous variables were expressed as median values and range. Intergroup differences in categorical variables were expressed as numbers and percentages. The chi-square statistical method was used for testing relationships between categorical variables. Linear regression models were used to determine associations between score change (lumbar BMD T-score, femoral neck BMD T-score, FRAX^®^ 10-year probabilities of major osteoporotic and hip fractures, probabilities defined by the Garvan tool, which is available online at https://www.garvan.org.au/promotions/bone-fracture-risk/calculator/index.php [16] accessed on 6 October 2021), and GIO prevention (bisphosphonate and active vitamin D). Statistical adjustments were implemented to account for potential confounding variables associated with the exposure and outcome. A sensitivity analysis was conducted to determine whether the results differed with and without adjustment for intravenous contrast. All tests were two-sided, 95% confidence intervals were calculated, and *p* < 0.05 was considered statistically significant. Analyses were conducted using Stata Version 14 (Stata Corporation, College Station, TX, USA), EZR (Saitama Medical Center, Saitama, Japan; http://www.jichi.ac.jp/saitama-sct/SaitamaHP.files/statmedEN.html accessed on 5 October 2021) [17], a graphical user interface for R (The R Foundation for Statistical Computing, version 2.13.0; www.r-project.org accessed on 5 October 2021), and a modified version of R commander (version 1.6-3) designed to add statistical functions.

## 3. Results

### 3.1. Patient Characteristics

Overall, 22 patients with ITP met the inclusion criteria in our institute between October 2014 and July 2021. The baseline characteristics of the patients are presented in Table 1. Antinuclear antibody was positive in four patients, all of which were not considered to have an autoimmune disease. Two other patients had an autoimmune disease (Sjögren’s syndrome (*n* = 1) and rheumatoid arthritis (*n* = 1)) before the diagnosis of ITP. *Helicobacter pylori* antibody was positive in six patients, all of which had received successful *Helicobacter pylori* eradication therapy before the diagnosis of ITP.

### 3.2. Treatment Response to the Initial Prednisolone Treatment

In our institution, the initial prednisolone treatments were used for patients presenting with a platelet count of <20 × 10^9^/L and bleeding symptoms. Patients with an indication for initial prednisolone treatment received a total median dose of 46 (range, 34–50) g/kg prednisolone and a median dose of ≥5 mg prednisolone daily over an administration period of 21 (range, 2–31) months. Seven patients achieved CR. Fifteen patients with refractory ITP received TPO-RA and 9 out of 15 patients achieved CR. The remaining six patients were defined as having a response (any platelet count between 30 and 100 × 10^9^/L and at least a doubling of the baseline count without bleeding symptoms). The median follow-up period was 26 (range, 8–86) months.

### 3.3. Score Changes during the Initial Loading of Prednisolone Treatment

Score changes during the initial loading of prednisolone treatment are summarized in Table 2. A study of the associations between score change and treatment of lumbar BMD T-score, femoral neck BMD T-score, FRAX^®^ 10-year probabilities of major osteoporotic and hip fractures, and probabilities defined by the Garvan tool, showed bisphosphonate prescription was significantly associated with an increase in the lumbar and femoral neck BMD T-score (*p* = 0.01, Table 3). Active vitamin D prescription was significantly associated with a low level of serum 25(OH)D at tapering phases of the course of prednisolone treatment (*p* < 0.01). There were no incidents of fracture during the initial prednisolone treatment period.

## 4. Discussion

Previous studies reported that 30–50% of adults receiving long term glucocorticoids developed fragility fractures [18]. BMD loss is rapid in the first few months of glucocorticoid use but continues to decline at a slower rate with continued use [11]. GIO is caused by multiple, complex mechanisms, including the suppression of bone formation by inhibiting osteoblast function, promoting osteoblast and osteocyte apoptosis [19], the inhibition of intestinal calcium absorption, and the reduction in gonadal hormones [20]. At our institute, we assessed DXA and started GIO prevention guided by femoral neck BMD T-score assessment. Although the median observation period was much shorter than 10 years, which is the anticipated observation period for FRAX^®^ and the Garvan tool, this early GIO prevention might reduce the risk of fragility fractures.

In this study, we analyzed the GIO in ITP patients aged ≥70 years according to femoral neck BMD T-scores < −1.0 measured by DXA scan and their association with GIO prevalence during the initial loading of prednisolone treatment. We found that the FRAX^®^ 10-year probabilities of major osteoporotic and hip fractures and probabilities defined by the Garvan tool were worse during the initial loading of prednisolone treatment compared with those during the tapering phases, but there were no incidents of fracture, and no significant differences in BMD loss or probabilities defined by FRAX^®^ and the Garvan tool between patients with and without bisphosphonate treatment. These results support our hypothesis that fractures in the early period of initial prednisolone treatment can be prevented by prescribing bisphosphonate guided by femoral neck BMD T-score assessment. Because 2 weeks of prednisolone treatment did not cause fractures in our patients, we could not investigate potential clinically significant differences in the probabilities defined by FRAX^®^ and the Garvan tool between the initial loading and the tapering phases of the prednisolone treatment.

Our data show changes in the lumbar BMD score, femoral neck BMD T-score, FRAX^®^ 10-year probabilities of major osteoporotic and hip fractures, probabilities defined by the Garvan tool, and bisphosphonate prescription were associated with an increased femoral neck BMD T-score. However, there were no incidents of fracture and no significant differences in BMD loss. This was attributed to the fact that early bisphosphonate treatment for ITP patients receiving high-dose prednisolone was associated with an increased likelihood of BMD loss. In the United States, hip fracture rates among persons aged 65 years and older are declining, and comorbidities among patients with hip fractures have increased [21], but there are no data on GIO in ITP patients aged ≥ 70 years. Further study after prospective long-term observation is needed to assess the effect of bisphosphonate on preventing fragility fractures in elderly ITP patients undergoing high-dose prednisolone therapy.

Our study found that active vitamin D prescription was significantly associated with a low level of serum 25(OH)D at tapering phases during prednisolone treatment. In Japan, sufficient vitamin D status reflected by serum 25(OH)D levels was associated with low limb and vertebral fracture risk in community-dwelling elderly women [22]. According to these data, we added active vitamin D for women, but not men. Our study did not demonstrate any benefit of the additional prescription of active vitamin D for elderly women with ITP undergoing prednisolone treatment, but the need for active vitamin D prescription for men is also unknown.

In our patient cohort, 15 patients received TPO-RA and switching from prednisolone to TPO-RA had a positive effect on the response and tolerability of patients aged ≥ 70 years. Prednisolone remains the initial treatment for newly diagnosed ITP patients [6]. Prednisolone has multiple effects on platelets including decreasing platelet clearance [23], increasing platelet production [24], and reducing bleeding independent of the platelet count increase, via a direct effect on blood vessels [25,26]. Although a consensus panel suggested that some patients were able to maintain a platelet response with a daily dose of <5 mg of prednisolone, the side effects of prednisolone outweigh their benefits over the long term [27]. The most appropriate management of ITP patients refractory to the initial treatment remains controversial. Although many drugs are available for refractory ITP, including TPO-RA and rituximab, as well as treatments including splenectomy, immunosuppressants, and novel therapies, individualized management is usually based on patient preference, side effects, previous treatment received, comorbidities, and cost associated with the treatment. Further studies are needed to assess the timing of switching from prednisolone to TPO-RA in elderly patients with refractory ITP according to the risk-adapted approach to GIO prevention.

This cohort study had some limitations, including its single institute nature. Although serum 25(OH)D levels before prednisolone treatment and active vitamin D administration should be accurately measured, serum 25(OH)D measurements are only permitted according to the rules of the Japanese government and health authorities. We added active vitamin D to the prescription for all women who received bisphosphonate and measured serum 25(OH)D levels in all patients to evaluate the treatment at the tapering phases of the course of prednisolone treatment. In addition, subjective data such as the assessment timing of BMD assessment were variable because this information depended on past medical records written by physicians before the study was planned. To minimize bias, we limited the inclusion criteria to patients who had at least one DXA assessment during the initial loading of prednisolone treatment at a single institution. The usage of FRAX^®^ is limited to those aged 40–75 years, but the FRAX^®^ in patients aged ≥75 years did not seem to be significantly different in clinical practice compared with other tools [28]. We assessed patient clinical information but did not determine morphometric vertebral fractures on the basis of medical records. Furthermore, a decrease in the initial prednisolone treatment intensity may have caused worse treatment outcomes in elderly patients. We selected patients who received TPO-RA at non-CR in the tapering phases of the course of prednisolone treatment because the effects of different second therapies for ITP according to the recommendations in the guidelines were unclear. Thus, there was potential for selection bias because no proper randomization could be achieved. Future research should apply standard prednisolone-dosing regimens, report patient-reported outcomes, and include cost-analysis evaluations, and the study design should include patients that require the extended use of steroids. Finally, the sample size, selection bias, especially that related to the exclusion of patients without prednisolone treatment because of difficulties in evaluation, and short follow-up duration may have limited our ability to analyze the outcomes and fractures of patients at tapering phases throughout prednisolone treatment. Although one consequence of these situations is the evident presence of multiple statistical comparisons that might have influenced some of the results obtained, the adequacy of the levels of statistical significance to the number of comparisons made and their dependence on the sample size indicate the conclusions should be considered with caution.

In conclusion, we might prevent GIO in elderly ITP patients using bisphosphonate treatment according to <−1.0 femoral neck BMD T-score measured by DXA scan. This study adds to information from previous studies on the real-world data of GIO in elderly patients with immune thrombocytopenia receiving prolonged steroid therapy. Prescribing bisphosphonate during the initial loading of prednisolone treatment might prevent the reduction in BMD and the additional prescription of active vitamin D for women might prevent fragility fractures, but the additional prescription of active vitamin D for women is still controversial. Further studies are needed to assess the timing of switching from prednisolone to a TPO-RA in elderly patients with refractory ITP according to the risk-adapted approach to GIO prevention.

## Figures and Tables

**Table 1:** Characteristics of patients with immune thrombocytopenia.

**Characteristic**		***n*** **= 22**
**Median age (range), years**		**79 (70–90)**
**>85 years old, *n* (%)**		**7 (32)**
**Body mass index, median (range), kg/m^2^**		**23 (17–31)**
**Sex, *n* (%)**	**male**	**7 (32)**
	**female**	**15 (68)**
**Antinuclear antibody positive, *n* (%)**		**4 (18)**
**Autoimmune disease, *n* (%)**		**2 (9)**
**History of *Helicobacter pylori* eradication, *n* (%)**		**6 (27)**
**Baseline platelet level (×10^9^/L), median (range)**		**16 (1–19)**
**Total prednisolone dose for treatment until** **<** **5 mg prednisolone daily administration, median (range), g/kg**		**46 (34–50)**
**≥** **5 mg prednisolone daily administration period, median (range), months**		**21 (2–31)**
**Thrombopoietin receptor agonist administration,** ***n*** **(%)**		**15 (68)**
**Bisphosphonate prescription, *n* (%)**		**15 (68)**
**Active vitamin D prescription, *n* (%)**		**11 (50)**
**Serum 25-hydroxyvitamin D post-prednisolone treatment, median (range), ng/mL**		**30.5 (17.0–34.0)**
**Follow-up time, median (range), months**		**26 (8–86)**

**Table 2:** Score change during the initial prednisolone administration.

Characteristic, Median (Range)		*n* = 22	OR ^a^	95% CI	*p*
**Lumbar BMD T-score**	**initial loading**	**−1.1 (−1.6–0.3)**	**reference**		
	**tapering phases**	**−1.3 (−1.8–−0.3)**	**0.46**	**−0.05–0.98**	**0.07**
**Femoral neck bone BMD T-score**	**initial loading**	**−1.1 (−1.6–0)**	**reference**		
	**tapering phases**	**−1.5 (−2.0–0)**	**0.23**	**−0.21–0.59**	**0.29**
**FRAX^® b^ (major osteoporotic, %)**	**initial loading**	**11.5 (5.4–23.0)**	**reference**		
	**tapering phases**	**12.0 (6.0–21.0)**	**0.94**	**0.86–0.97**	**<0.01**
**FRAX^® b^ (hip, %)**	**initial loading**	**3.1 (0.8–7.8)**	**reference**		
	**tapering phases**	**3.5 (0.6–8.7)**	**0.84**	**0.66–0.93**	**<0.01**
**Garvan-defined osteoporotic fracture** **(5 year, %)**	**initial loading**	**9 (4–27)**	**reference**		
	**tapering phases**	**9.5 (4–31)**	**0.89**	**0.76–0.95**	**<0.01**
**Garvan-defined osteoporotic fracture** **(10 year, %)**	**initial loading**	**18 (7–45)**	**reference**		
	**tapering phases**	**19 (8–51)**	**0.91**	**0.79–0.96**	**<0.01**
**Garvan-defined hip fracture** **(5 year, %)**	**initial loading**	**2 (0.2–5)**	**reference**		
	**tapering phases**	**2 (0.4–11)**	**0.83**	**0.65–0.93**	**<0.01**
**Garvan-defined hip fracture** **(10 year, %)**	**initial loading**	**3 (0.4–10)**	**reference**		
	**tapering phases**	**4 (0.7–20)**	**0.85**	**0.68–0.94**	**<0.01**

Initial loading, initial loading of the course of prednisolone treatment; tapering phases, tapering phases of the course of prednisolone treatment; OR, odds ratio; CI, confidence interval; BMD; bone mineral density. ^a^ Odds ratios indicate the likelihood of a percentage or score per reference. ^b^ FRAX^®^ 10-year probability of major osteoporotic or hip fractures is calculated.

**Table 3:** Association between the prevention of glucocorticoid-induced osteoporosis and score change.

	with Bisphosphonate			with Active Vitamin D		
Characteristic	c.e.	95%-CI	*p*	c.e.	95%-CI	*p*
**Lumbar BMD** **T-score**	**0.61**	**0.14–1.08**	**0.01**	**0.36**	**−8.77–9.50**	**0.93**
**Femoral neck bone BMD T-score**	**1.05**	**0.24–1.86**	**0.01**	**−0.37**	**−1.13–0.37**	**0.30**
**FRAX^® a^ (major osteoporotic, %)**	**−1.81**	**−4.13–0.51**	**0.11**	**1.87**	**−0.29–4.03**	**0.08**
**FRAX^® a^ (hip, %)**	**−1.36**	**−3.12–0.39**	**0.12**	**1.26**	**−0.37–2.90**	**0.12**
**Garvan-defined osteoporotic fracture (5 year, %)**	**−2.39**	**−7.88–3.10**	**0.37**	**2.43**	**−2.68–7.54**	**0.33**
**Garvan-defined osteoporotic fracture (10 year, %)**	**−4.82**	**−13.0–3.42**	**0.23**	**4.70**	**−2.97–12.3**	**0.21**
**Garvan-defined hip fracture (5 year, %)**	**−1.77**	**−4.26–0.70**	**0.15**	**1.35**	**−0.96–3.66**	**0.23**
**Garvan-defined hip fracture (10 year, %)**	**−3.13**	**−7.66–1.40**	**0.16**	**2.56**	**−1.66–6.79**	**0.22**

c.e., coefficient; CI, confidence interval; BMD, bone mineral density. ^a^ FRAX^®^ 10-year probability of major osteoporotic or hip fractures is calculated.

## Data Availability

The institutional review board of Kyushu University Hospital, Japan, does not allow open access. However, on reasonable request, additional analyses can be performed after contacting the corresponding author.
